# The implementation of a robotic surgical platform for the treatment of patients with malignant or pre-malignant pancreatic tumors at the University Medical Center Ljubljana

**DOI:** 10.2478/raon-2025-0051

**Published:** 2025-09-05

**Authors:** Miha Petric, Patricio Marcelo Polanco, Jan Grosek, Ales Tomazic, Blaz Trotovsek, Bostjan Plesnik

**Affiliations:** Department of Abdominal Surgery, University Medical Center Ljubljana, Ljubljana, Slovenia; Medical Faculty, University of Ljubljana, Ljubljana, Slovenia; Division of Surgical Oncology, UT Southwestern Medical Center, Dallas, TX, USA

**Keywords:** robotic pancreatic surgery, implementation, minimal invasive surgery

## Abstract

**Background:**

Robotic platforms are increasingly employed in the field of minimally invasive pancreatic surgery. It is essential to develop an innovative method that ensures both safety and efficacy, producing outcomes comparable to those of established treatment modalities. Implementation process should incorporate surgical science, education, local implementation, and non-technical skills. In our study, we describe the safe implementation of a robotic platform in pancreatic surgery within our medical institution.

**Patients and methods:**

We analysed prospectively collected data from the first ten consecutive robotic-assisted distal pancreatectomies (RDP) and pancreatoduodenectomies (RPD). Due to nature of the study basic statistical analysis were performed.

**Results:**

The mean operating time was 211minutes (±49.4) for RDP and 365 minutes (±69.6) for RPD, with blood loss 330 mL for RDP and 195 mL for RPD. Hospital stay was 8.7 days (±3.9) in RDP and 7.9 days (±3.9) in RPD. One patient (10%) in the RDP group developed clinically relevant postoperative pancreatic fistula (CR-POPF) and delayed gastric emptying (DGE). The mean tumour size was 31 mm (±9.8) in the RDP and 27 mm (±7.5) in the RPD. The mean number of lymph nodes harvested was 6 (0–24) in the RDP and 15 (6–22) in the RPD. The R0 resection rate was 60% in the RDP and 70% in the RPD.

**Conclusions:**

Robotic surgical technology can be safely and effectively integrated into a clinical setting. This integration should be facilitated through a well-established training program and curriculum. Nonetheless, patient selection is important, especially in the early phases of robotic program development.

## Introduction

In recent years, robotic surgical platforms have been increasingly used in the field of pancreatic surgery.^1^ Evidence from the literature suggests that these robotic platforms enhance the safe and effective management of patients with malignant or premalignant pancreatic conditions.^2^ Initial reports from pioneers in robotic pancreatic surgery indicated steep and demanding learning curves, requiring between 100 to 200 cases^3,4^ to achieve competency in pancreatoduodenectomies and approximately 40 cases^4,5^ for distal pancreatectomy. Currently, established surgical and learning protocols facilitate the more rapid implementation of robotic platforms in pancreatic surgery.^6^ However, despite the increasing availability of these robotic platforms, it is imperative for surgeons to prioritize patient safety and well-being. Open pancreatic surgery has a well-established history dating back to the early 20th century.^7^ With advancements in surgical knowledge and technology, enhanced perioperative management has been linked to relatively low mortality rates (3–5%)8,9 and still relatively high morbidity rates (40%–60%).8,9 The integration of systemic therapy has facilitated long-term survival for a significant proportion of patients undergoing surgery for pancreatic adenocarcinoma.^10^ It is essential that new surgical techniques are integrated into routine practice with meticulous planning and a step-by-step approach. This study aimed to demonstrate the successful implementation of a robotic surgical platform at a high-volume tertiary medical center.

## Patients and methods

An analysis of prospective collected data was conducted on the initial ten cases of robot-assisted left hemipancreatectomies and pancreatoduodenectomies performed at the Department of Abdominal Surgery, University Medical Center, Ljubljana, commencing in June 2022. We collected data on patients’ general characteristics, including age, sex, BMI, aetiology of pancreatic disease, and ASA score. Intraoperative parameters, such as the duration of the surgical procedure, blood loss, conversion, pancreatic duct measurement, pancreatic gland constitution, and use of drains, were also recorded. Postoperative parameters included the length of hospitalization, postoperative complications classified according to the Clavien-Dindo classification, 30-day readmission rate, and 90-day mortality rate. Additionally, we gathered data on oncological outcomes post-surgery, including tumour histology, the number of retrieved and positive lymph nodes, radicality of resection (R0/R1), adjuvant chemotherapy, and the two-year survival rate.

The study adhered to the principles of Good Clinical Practice and the tenets of the Declaration of Helsinki. A detailed explanation was provided, and written informed consent was obtained from each patient prior to the procedure.

### UT Southwestern robotic curriculum program

The MP visited the UT Southwestern Medical Centre at the University of Texas and successfully completed a proficiency-based training program in robotic surgery.^11^ The program comprised an eightweek intensive training, beginning with an initial video assessment and evaluation during three virtual reality (VR) tasks and two inanimate drills. Subsequently, 20 designated VR tasks were completed until a total score of ≥ 90% was achieved.^12^ Successful completion of the VR component was followed by inanimate tasks, which were evaluated using the Objective Structured Assessment of Technical Skills (OSATS).^13^ This was succeeded by bio-tissue drills, during which gastro-jejunal, hepatico-jejunostomy, and pancreatico-jejunal anastomoses were performed and assessed using OSATS metrics.^14^ Throughout the training program, the MP was granted access to a video library, and observed live surgeries. Upon completing the training program, the MP commenced robot-assisted cholecystectomy and splenectomy under the supervision of an experienced robotic surgeon (Primož Sever, Simon Hawlina). The first robot-assisted distal pancreatectomy was performed in June 2022, followed by nine consecutive robot-assisted distal pancreatectomies. The inclusion criteria for robotic distal pancreatectomy comprised the presence of a tumor located in the left portion of the pancreas, with no indications of portal vein infiltration. The exclusion criteria included signs of portal vein infiltration, evident infiltration of adjacent organs such as the stomach or colon, or a history of major upper gastrointestinal surgery, including gastric or liver surgery. Under PMP proctorship, the first robot-assisted pancreatoduodenectomy was conducted in November 2022. The criteria for inclusion in robotic pancreatoduodenectomy required the presence of a tumor located in the pancreatic head, along with a dilated main pancreatic duct (greater than 5 mm) and hepatic duct (greater than 10 mm), and the absence of portal vein infiltration. The exclusion criteria encompassed evidence of portal vein infiltration, a narrow pancreatic duct (less than 5 mm) or hepatic duct (less than 10 mm), a body mass index (BMI) exceeding 35, or a history of major upper gastrointestinal surgery, such as gastric or liver surgery. Notably, after the fourth case, a narrow pancreatic or bile duct was no longer considered an exclusion criterion. BP successfully completed the same structured learning program at UT Southwestern in 2024.

### Standard operative protocol

For all robotic-assisted procedures, the da Vinci Xi robotic platform was used. We aimed to standardize all surgical steps and procedures with the education of all surgical team members, including the anesthesiology team and scrub nurses.

#### Robotic assisted approach to left pancreas

**FIGURE 1.**
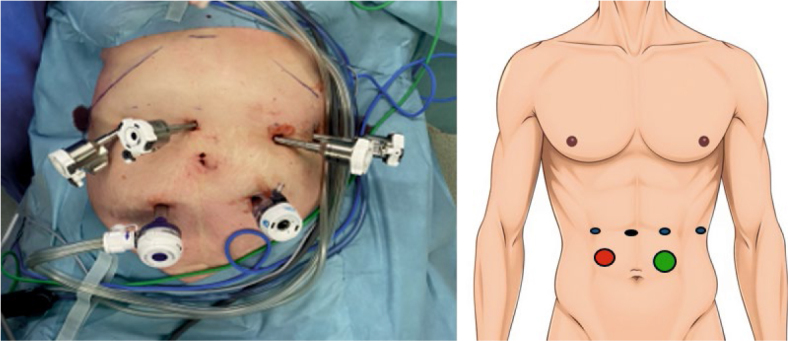
trocars position for robotic assisted distal pancreatectomy; 8mm robotic arm ports (blue), 8 mm robotic camera port (black), AirSeal® port (red), 15 mm assistant port (green).

We adhered to the standard operative protocol established by the UT Southwestern group^15^, incorporating minor modifications. The patient underwent endotracheal intubation and was placed in the French position. If adequate peripheral venous access was achievable, a central venous line was deemed unnecessary. The patient was secured, and pressure points were padded to prevent pressure-induced injury to the skin and tissue. Urinary catheter was inserted. Prior to robotic docking, the patient was positioned in a 15-degree anti-Trendelenburg position with a 5-degree tilt to the right. A skin incision was made at the anticipated location of the robotic camera arm, and a pneumoperitoneum of 12 mmHg was established. Robotic 8 mm arm port was inserted. Laparoscopy of the abdominal cavity was conducted to rule out distant metastases and signs of inoperability. The positions for three additional 8 mm robotic arm ports, 12 mm AirSeal® port, and a 15 mm assistant port were marked and inserted under direct vision (Figure 1).

A Mediflex ® FlexArm ™ retractor was employed for gastric retraction. Initially, the lesser sac was opened, and the pancreatic gland was examined. In cases where tumours were not visible, intraoperative ultrasound was utilized. The hepatic artery approach facilitated the removal of lymph node station 8a, and the portal vein was dissected. Subsequently, the hepatic artery, truncus coeliacus, along with the splenic artery were exposed. The next step involved the dissection of the gastrocolic ligament using LigaSure™ (Medtronic) at the greater gastric curvature above the gastroepiploic arcade, followed by the dissection of the left gastrocolic pedicle and mobilization of the lienal flexure to expose Gerota’s fascia. Subsequently, short gastric veins were transected using LigaSure™ (Medtronic). In cases where spleen preservation was indicated, this step was omitted. A retractor was employed for optimal gastric retraction. We dissected the origin of the lienal artery and then closed and resected the lienal artery (LA) between two proximal and one distal Hem-o-lok® polymer clips. The subsequent step involved the mobilization of the lower pancreatic aspect medial to the tumour and exposure of the confluence of the renal and superior mesenteric veins. Dissection and visualization of the portal vein were performed. The location of the pancreatic lesion site for pancreatic gland transection was determined. In cases of a soft or steatotic pancreas, Endo GIA™ with a reinforced mesh was utilized. If the pancreatic gland was firm, transection was performed with monopolar robotic scissors, and the pancreatic duct was closed using a PDS 5-0 suture. The pancreatic stump was oversewn with a 2-0 Silk suture. In cases of malignant tumours, anterior radical antegrade modular pancreatosplenectomy (RAMPS) was executed. Spleen-preserving left/distal pancreatectomy was performed for benign and premalignant lesions. The distal stump of the pancreas was retracted with robotic arm 4, and short veins originating from the splenic vein were dissected with LigaSure™ (Medtronic). Branches of the lienal artery were clipped at their origin with a Hem-o-lok® polymer clips and cut distally with LigaSure™ (Medtronic). Finally, the specimen was placed in an Endo Bag™ (Medtronic) and extracted through a Pfannenstiel incision. An intraoperative frozen section was obtained if indicated, and an intra-abdominal drain was placed through robotic trocar no. 1 or 4 and securely fixed.

#### Robotic assisted approach to pancreatic head resection

**FIGURE 2.**
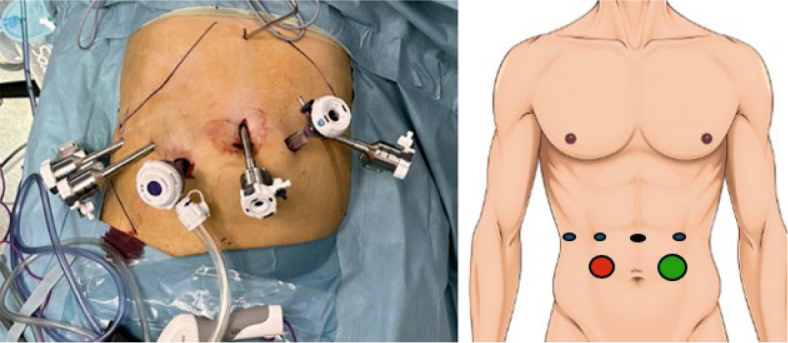
trocars position for robotic assisted distal pancreatectomy; 8mm robotic arm ports (blue), 8 mm robotic camera port (black), AirSeal® port (red), 15 mm assistant port (green).

We adhered to the standard operative protocol established by the UT Southwestern group, incorporating minor modifications. The patient underwent endotracheal intubation and a central venous line was inserted. The patient was secured in the French position and pressure points were added to prevent pressure-induced injury to the skin and tissue. Urinary catheter was inserted. Prior to robotic docking, the patient was positioned in a 15-degree anti-Trendelenburg position with a 5-degree tilt to the left. A skin incision was made at the anticipated location of the robotic camera arm, and a pneumoperitoneum of 12 mmHg was established. An 8 mm robotic arm port was inserted. Laparoscopy of the abdominal cavity was performed to rule out distant metastases and any signs of inoperability. The positions of three additional 8 mm robotic arm ports, a 12 mm AirSeal® port, and a 15 mm assistant port were marked and inserted under direct vision (Figure 2).

An Mediflex ® FlexArm ™ retractor was employed to retract the liver. We followed steps, as described by Gulianoti *et al*.^16^ and UT Southwestern SOP, with slight modifications to the method. These steps included opening the gastrocolic ligament and exposing the pancreas, takedown of the right colonic flexure to the level of the lower pole of the right kidney, Kocher manoeuvre with transection of the first jejunal loop (using an Endo GIA™), division of the right gastric artery (using a Hem-o-lok® polymer clips), division of the duodenum (using an Endo GIA™), exploration and lymphadenectomy from truncus coeliacus to the hepatic hilum, retrograde cholecystectomy, common bile duct transection above the insertion of the cystic duct, division of the gastroduodenal artery (GDA) (using a Hem-o-lok® polymer clips), transection of the pancreatic neck (using monopolar scissors), exposure of the superior mesenteric vein and division of the right gastroepiploic artery (using a Hem-o-lok® polymer clips), exposure of the first jejunal vein and dissection of the uncinate process, pancreatico-jejunostomy (modified Blumgart anastomosis^17^), hepatico-jejunostomy reconstruction (using interrupted PDS 5-0), and duodeno-jejunal anastomosis (using a single continuous self-locking 4-0 suture). Tissue transection was done using monopolar hook and LigaSure™ (Medtronic). Finally, the specimen was placed in an Endo Bag™ (Medtronic) and extracted via a Pfannenstiel incision. An intraoperative frozen section was obtained if indicated, and an intra-abdominal drain was placed through robotic trocar no. 1 and securely fixed.

### Statistical analysis

Data were analysed using SPSS for macOS, 26th edition. Descriptive statistics such as frequencies, percentages, mean/median, and standard deviations were used for description and summary. Because the data were not normally distributed, patient characteristics were compared between groups using the Mann-Whitney U test. A P-value ≤0.05 was considered statistically significant.

## Results

Beginning in June 2022, we conducted a series of 10 consecutive robot-assisted left hemipancreatectomies (RDP) and 10 pancreatoduodenectomies (RPD) within the Department of Abdominal Surgery. The baseline demographic and clinical characteristics of the study population are detailed in Table 1.

**TABLE 1.** Baseline characteristics of the study population

	RDP (n = 10)	RPD (n = 10)
**Gender, males (%)**	20	60
**Mean age, years (±SD)**	61.8 (±7.6)	70.5 (±8.6)
**Mean BMI, kg/m^2^ (±SD)**	29.3 (±6.0)	26.2 (±4.0)
**ASA score, n (%)**		
**I**	0 (0%)	0 (0%)
**II**	5 (50%)	4 (40%)
**III**	5 (50%)	6 (60%)
**IV**	0 (0%)	0 (0%)
**V**	0 (0%)	0 (0%)
**Histology**		
**PDAC**	4 (40%)	7 (70%)
**IPMN**	3 (30%)	1 (10%)
**NEN**	2 (20%)	1 (10%)
**MCN**	1 (10%)	0 (0%)
**GIST**	0 (0%)	1 (10%)

1 ASA = American Society of Anesthesiology, BMI = body mass index; GIST= gastrointestinal stromal tumor; IPMN = intraductal papillary mucinous neoplasm; MCN = mucinous cystic neoplasm; NEN = neuroendocrine neoplasm; PDAC = pancreatic ductal adenocarcinoma; RDP = robotic-assisted distal pancreatectomy, RPD = robotic-assisted pancreatoduodenectomy, SD = standard deviation

### Operative outcomes

Operative outcomes are detailed in Table 2. One patient undergoing RDP required conversion to open surgery due to intraoperative suspicion of malignant infiltration of the coeliac trunk and mesocolon. Following open exploration, a total pancreatectomy and segmental resection of the portal vein were performed.

**TABLE 2.** Perioperative outcomes

	RDP (n = 10)	RPD (n = 10)
**Conversion**	1(10%)	0
**Mean operative time (min)**	211 (±49.4)	365 (±69.6)
**Intraoperative blood loss (ml)**	330 (100 – 1700)	195 (100 - 500)
**Pancreatic texture**		
**Soft**	9 (90%)	3(30%)
**Hard**	1(10%)	7(70%)
**Spleen preserving (Kimura)**	3 (30%)	n/a
**MPD diameter in mm(range)**	n/a	6 (2-8)
**BD diameter in mm (range)**	n/a	9(5-14)
**POPF**		
**Leak**	2(20%)	3 (30%)
**B**	1 (10%)	0
**C**	0	0
**DGE**	0	1(10%)
**Blood transfusion**	3 (30%)	2(20%)
**30-readmision rate**	1 (10%)	1(10%)
**90-day mortality rate**	0	0

1 BD = bile duct; DGE = delayed gastric emptying; MPD = main pancreatic duct; POPF = postoperative pancreatic fistula; RDP = robotic-assisted distal pancreatectomy; RPD = robotic-assisted pancreatoduodenectomy

**FIGURE 3.**
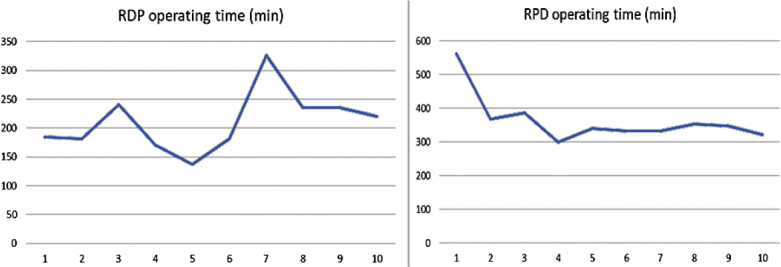
Operating time. RDP = robotic-assited distal pancreatectomy; RPD = robotic-assisted pancreatoduodenectomy

Higher volume of intraoperative blood loss in RDP group is contributed to conversion case (1200 mL). The mean operative time was 211 minutes (±49.4) for RDP and 365 minutes (±69.6) for RPD. In the RPD group, a continuous improvement in operative time was observed, whereas in RDP, the operative time remained more consistent, likely due to the early achievement of the learning curve (Figure 3.)

### Postoperative outcomes

All patients were admitted to the intensive care unit (ICU) postoperatively. The mean ICU stay was 3.4 days (±2.7) for RDP patients and 2.8 days (±1.2) for RPD patients. The overall length of hospital stay was comparable: 8.7 days (±3.9) for RDP and 7.9 days (±3.9) for RPD. Postoperative complications are listed in Table 2. The 30-day readmission rate was 10% in both groups. One patient in the RDP group was readmitted due to type B postoperative pancreatic fistula (POPF), which was effectively managed with endoscopic trans-gastric (AXIOS™ Stent) drainage. Another patient in the RPD group was readmitted because of a bleeding marginal ulcer and subsequent iatrogenic perforation during the endoscopy, necessitating open surgical intervention. No postoperative mortality was observed within 90 days.

### Pathology reports

The mean tumor size was 31 mm (±9.8) in the RDP group and 27 mm (±7.5) in the RPD group. General report on group pathology is shown in Table 1. The mean number of lymph nodes harvested during RDP was 6 (range of 0 to 24), although two reports did not include lymph node data, one of which notably involved splenectomy. In contrast, the RPD group had a mean of 15 lymph nodes retrieved (range of 6 to 22). The R0 resection rate was 60% in the RDP group and 70% in the RPD group. All R1 resection margin involved posterior pancreatic margin.

### Adjuvant therapy and long-term outcomes

All four RDP patients diagnosed with PDAC received adjuvant chemotherapy: three received mFOLFIRINOX, and one received gemcitabine. In the RPD group, four of six eligible PDAC patients received adjuvant chemotherapy: three received gemcitabine and one mFOLFIRINOX.

The two-year survival rate was 90% after RDP and 50% following RPD. In all instances, the cause of death was the systemic progression of PDAC. The incidence rates of postoperative incisional hernia were 10% and 30% after RDP and RPD, respectively.

### Comparison of robotic to open pancreatoduodenectomy

We conducted a comparative analysis of the initial 10 robotic pancreatoduodenectomies (RPD) and the most recent 35 open pancreatoduodenectomies (OPD) performed by MP (Table 3.).

**TABLE 3.** Comparison of robotic and open pancreatoduodenectomy

	RPD	OPD	P-value
**male**	6	21	P=0.50
**female**	4	14	
**AGE**	72 (59-83)	71	P =0.65
**BMI**	26 (20,5- 33,3)	26	P=0.48
**1**	0	0	
**2**	1	9	
**3**	9	26	
**4**	0	0	
**Time OFOP**	364 {300- 542)	216 (163- 322)	P<0.0001
**Conversion to open**	0	np	
**Texture of pan**			
**Soft**	3 (30%)	18 (51,4)	P=0.12
**Hard**	7(70%)	17 (48,6%)	
**Blood loss in ml**	205 (100- 500)	491 (200- 700)	P<0.0005
**HOU (0)**	2,7 (1-5)	7(3- 92)	P =0.07
**Hospitalisation (0)**	7,9 (5-19)	15,2(7-133)	P= 0.05
**CR -POPF**	0	9 (25%)	P=0.006
**OGE**	1 (10%)	6 (17%)	P= 0.42
**300 Rehospitalisation**	1 (10%)	3 (8,6%)	P=0.17
**900 Mortality**	0	0	
**Histology**			
**Premalignant**	1 pts (10%)	5 (14,3%)	
**Malignant**	9 pts (90%)	30(85,7%)	
**Raoicality**			
**RO**	7/10 (70%)	30/35(85,7%)	P=0.13
**n**	15,5 (9-19)	19 (2-48)	P=0.12

1 ASA = American Society of Anesthesiology; BMI = body mass index; CR-POPF = clinically relevant postoperative pancreatic fistula; DGE = delayed gastric emptying; HDU = high dependency unit; op = operation; OPD = open pancreatoduodenectomy; PAN = pancreas; RPD = robotic-assisted pancreatoduodenectomy

The RPD group demonstrated a significantly longer operative duration (P < 0.0001), shorter hospitalisation (P = 0.05) and reduced blood loss (P < 0.0005). The incidence of clinically relevant postoperative pancreatic fistula (P = 0.006) was notably lower in the RPD cohort. No difference was observed in the rate of R0 resection (P = 0.13) or lymph node retrieval (P = 0.12) between the two groups. However, it is important to consider the potential bias introduced by patient selection in RPD group and case numbers when interpreting these results of this study.

## Discussion

Our preliminary findings indicate that the robotic surgical platform can be safely integrated into our clinical setting. Additionally, we have demonstrated that the UT Southwestern standard operative protocols are reproducible and can be effectively implemented outside of a high-volume, specialized center.

Søreide^18^ has articulated a formula for survival in surgery, which encompasses surgical science, education, local implementation, and non-technical skills. Fernandes *et al*.^19^ demonstrated that robotic pancreatic surgery exhibits morbidity and mortality rates comparable to those of open surgery, with similar oncological outcomes. In 2019, the Miami International Evidence-based Guidelines on Minimally Invasive Pancreas Resection^20^ advocated for minimally invasive distal pancreatectomy in cases of benign and low-grade malignant tumors. Subsequently, the Brescia Internationally Validated European Guidelines on Minimally Invasive Pancreatic Surgery (EGUMIPS)^21^ refined these guidelines, offering clearer definitions of indications, patient selection criteria, surgical techniques, and training protocols. A systematic review and meta-analysis^22^ indicated that robot-assisted distal pancreatectomy is comparable to open or laparoscopic approaches concerning the rate of severe complications and the incidence of clinically relevant postoperative pancreatic fistula. This finding was corroborated by another metaanalysis, which confirmed that robotic resection can be safely executed irrespective of tumor location.^23^ Analysis of short-term surgical outcomes using large population data revealed that robotic pancreaticoduodenectomy (RPD) was associated with a lower incidence of postoperative complications (46.8% vs. 53.3%, P = 0.004) and surgical complications (42.6% vs. 48.6%, P = 0.008) compared to open pancreaticoduodenectomy (OPD).^24^ Long-term oncological outcomes, as analyzed by Da Dong *et al*., indicated that RPD was associated with a higher incidence of R1 resections (15.6% vs. 19.9%; OR (95% CI) = 0.64 (0.41, 1.00); p = 0.05) than OPD. Additionally, the number of retrieved lymph nodes was greater in the RPD group (MD (95% CI) = 2.88 (1.12, 4.65); p = 0.001).^25^ A seminal study from the Pittsburgh group demonstrated that, upon achieving proficiency, RPD can be associated with a low incidence of severe complications (CD > 2, 24%), clinically relevant postoperative pancreatic fistula (CR-POPF, 7.8%), a length of stay (LOS) of 8 days, and low 30- and 90-day mortality rates (1.4% and 3.1%, respectively).^26^ The robotic surgical platform demonstrates comparable efficacy to open surgery in the management of left-sided pancreatic disease, particularly concerning early surgical and oncological outcomes.^27^ The DIPLOMA trial^28^ established that minimally invasive distal pancreatectomy (MIDP) is non-inferior to open distal pancreatectomy (ODP) for pancreatic cancer. A comprehensive nationwide audit conducted by the Dutch Pancreatic Cancer Audit^29^ revealed that MIDP is associated with a reduced hospital stay (median 7 vs. 8 days, P < 0.001) and decreased blood loss (median 150 vs. 500 mL, P < 0.001), although it presents a higher incidence of clinically relevant postoperative pancreatic fistula (CR-POPF) (24.4% vs. 17.2%, P = 0.008) compared to ODP. Our findings are consistent with the extant literature concerning median operating times, which were 211 minutes (±49.4) for RDP and 365 minutes (±69.6) for RPD. Recorded blood loss was 330 mL for RDP and 195 mL for RPD. The length of hospitalization was 8.7 days (±3.9) for RDP and 7.9 days (±3.9) for RPD. On average, 6 (range: 0–24) lymph node were retrived in the RDP cohort and 15 (range: 6–22) in the RPD cohort. The conversion rate for RDP was relatively high at 10%, likely attributable to the limited number of cases. Similarly, the R0 resection rate was 60% for RDP and 70% for RPD, probably due to the low number of cases. There were no instances of mortality within the 90-day period.

In 2020, Vining *et al*.^30^ introduced a structured curriculum for training programs in minimally invasive pancreatic surgery. This curriculum comprises a preclinical training phase that includes platform familiarization, patient positioning, observation of video and operating procedures, virtual reality (VR) simulation, and bio-tissue drills.^30^ Participants who successfully complete the initial phase proceed to perform simple procedures under proctorship guidance, thereby entering the clinical training phase, where they achieve competence, proficiency, and mastery.^30^ In the second phase, the roles of proctoring and mentoring are essential for advancing the learning trajectory and ensuring patient safety.^31^ The integration of robotic VR simulation and intrinsic skills into residency curricula should be standard practice in centers equipped with robotic platforms, as it is associated with a high completion rate and excellent feasibility.^32^ Residents with early adoption of economy of motion skills may acquire robotic surgery skills more rapidly.^33^ Biotissue models provide enhanced haptic feedback and enable realistic suturing, both of which are essential for effective anastomosis training and, consequently, for improving patient outcomes.^34^ Early reports showed that surgeons with prior experience in open pancreatic surgery demonstrated superior performance compared to their inexperienced counterparts, both at the early stages of robotic program and in complex case scenarios.^35^ A recent review, despite the low quality of evidence in the existing literature, indicated that skill transfer is most beneficial when advanced robotic tasks are undertaken during the initial phase of the learning curve.^36^ MP, the lead surgeon in the robotic hepatopancreatobiliary (HPB) program, has over nine years of experience as a consultant in HPB and liver transplant surgery. This experience encompasses the performance of more than 120 open pancreatoduodenectomies and 30 liver transplantations in our institution. Prior to the establishment of the robotic pancreatic program, the annual surgical volume included an average of 20 open pancreatoduodenectomies and up to 5 distal pancreatectomies, most of which were performed laparoscopically. During this period, BP was a senior resident with substantial experience assisting with all HPB and liver transplant procedures. Data from recent literature indicate that incorporating a structured approach in the development phase of the robotic platform results in a learning curve for robotic pancreaticoduodenectomy (RPD) of between 40 and 80 cases^37–39^, and for robotic distal pancreatectomy (RDP) of up to 40 cases.^40,41^ Results from the LAELAPS-3 training program^42^ demonstrated that feasibility, proficiency, and mastery are achieved at 15, 62, and 84 RPD procedures^43^, respectively, which is significantly shorter than in previously reported series.^43^ The structured approach facilitates the effective and successful establishment of a safe robotic pancreatic program globally.^44–47^ Despite the relatively low number of cases required to complete the learning curve, robotic pancreatic surgery should be implemented at university or tertiary centers.^48^ Panni *et al*. found that performing 36 cases per year significantly correlates with improved surgical outcomes.^49^ The centralization of pancreatic surgery is associated with higher resection rates and better overall survival.^50^ The beneficial effects of centralization can be attributed not only to surgical management but also to the availability of multimodal approaches, particularly in the context of postoperative complications, as well as the knowledge and experience involved.^51^ In the RPD group, a continuous improvement in operative time was observed, whereas in the RDP group, the operative time remained more consistent, likely due to the early achievement of the learning curve. This consistency is probably attributable to the lead author’s prior expertise in open and laparoscopic approaches to left-sided pancreatic disease.

Lastly, non-technical skills, as identified by Soreide^18^, are among the most critical factors, yet they are frequently overlooked or absent. Skills such as situational awareness, decision-making, leadership, communication, and teamwork are essential parameters for the development of sustainable and successful surgical programs.^52^ Successful surgical programs can be established only through the integration of clinical excellence, ongoing education, research, diversity, and the acceptance of diverse ideas and methodologies.^53^

Our study acknowledges several limitations. A primary concern is the potential bias in patient selection. Specifically, candidates for robotic pancreaticoduodenectomy (RPD) were carefully chosen, excluding those with narrow pancreatic ducts, soft or steatotic glands, major vessel infiltration, or a high body mass index (BMI) from robotic-assisted surgery. Nevertheless, this selection process resulted in a successful initial series with outcomes comparable to open surgery. Currently (after 100 pancreatic resections), only a history of complex previous upper gastrointestinal procedures or indications of tumor invasion in major vessels serve as contraindications for the robotic-assisted approach.

In conclusion, we have demonstrated that novel surgical technology can be safely and effectively integrated into a clinical setting. This integration should be facilitated through a well-established training program and curriculum. We have shown that the UT Southwestern SOP^15,26^ can be adopted safely and efficiently, yielding results comparable to those of open or laparoscopic modalities. As a tertiary university center, we are now equipped to offer a robotic platform to patients with malignant or premalignant pancreatic diseases, serving as a complementary approach to traditional open or laparoscopic methods. Nonetheless, patient selection is important, especially in the early phases of robotic program development. Drawing upon the findings of our study and our experience with the implementation of a robotic surgical platform in pancreatic surgery, we intend to establish a curriculum for robot-assisted pancreatic surgery at UMC Ljubljana, modelled after the program at UT Southwestern.
